# Ten Simple Rules for Experiments’ Provenance

**DOI:** 10.1371/journal.pcbi.1004384

**Published:** 2015-10-20

**Authors:** Toni Kazic

**Affiliations:** Dept. of Computer Science Missouri Maize Center, Missouri Informatics Institute, and Interdisciplinary Plant Group, University of Missouri, Columbia, Missouri, United States of America

Everyone needs experimental data to understand biology. Exactly how and from what the data were obtained determines an experiment’s results, specifies how it can be reproduced, and conditions our analyses and interpretations. These details of materials, methods, and analyses are the experiment’s provenance.

Today, as it has been for hundreds of years, experimental provenance is recorded in some form of laboratory notebook. But as data migrate from the experimentalist’s mind and notebook to publication, the lab server, the archival database, or the cloud, this essential information now vanishes. Like interpretation, our ability to reproduce results depends on knowing how they were produced by others. Shorn of their immediate context, the methodological ideas and information that were perfectly transparent to the experimentalist (or computationalist!) become opportunities for error-prone reconstruction by others, even within the same group [1–3]. That reconstruction requires (repeated) private communications, rereading notebook entries, polling one’s own or a group’s collective memory, and looking at the specimens. None of those methods are reliable, and all are tedious.

As big data become a reality, it will be ever more imperative to encapsulate experimental provenance with the data. But how do we get that information out of the brains and notebooks in the first place? This is a problem of information capture, not data formats; of laboratory practice, not resource discovery; and of the million flowers of experimental creativity, not ontology building. Of course, ontologies, interoperable grid resources, and efficient search are important and appealing—but absent experimental provenance, they are biologically moot.

The obvious solution might seem to be standards for post hoc data annotation by biologists. Indeed, several previous efforts have defined sets of “minimal” metadata about particular types of high-throughput experiments, beginning with the minimal information needed for microarray experiments (the MIAME criteria) [4]. However, experience shows three fundamental problems with this approach. First, despite vigorous encouragement from computational biologists, most deposited datasets lack such annotation [1]. Second, the universe of experiments performed, let alone possible, far exceeds the stamina of even the most earnest committees to promulgate definitions and criteria. As always, science outruns nomenclature.

The third fundamental problem is even more basic than effort or invention. Many experimentally inclined biologists are too ill equipped and too busy to produce electronic provenance metadata in almost any form. Large experimental consortia and high-throughput facilities often do develop in-house provenance systems, and these and commercial ones are available (for example, see [5,6]). But smaller groups also generate essential data, often with a wide variety of experiments that don’t fit existing packages, standards, or ontologies. Their provenance information tends to be fragmented, buried in a mix of paper and electronic records, and dependent on the group’s institutional memory. Provenance is especially important in these contexts, since many of these experiments will nucleate the hypotheses and provide the materials for the subsequent high-throughput experiments that are the meat and potatoes of much of contemporary computational biology. While metadata are notoriously difficult to obtain from experimentalists, in principle they could be computed from adequate electronic provenance records. The first difficulty in that sentence is “adequate electronic provenance records.” Like other forms of housekeeping, it is easy for provenance to be mere drudgery, without the glass slipper at the end.

How can we make provenance easier so it is better for all? The trick is to capture provenance as the experiment is planned, performed, and analyzed. The easier, more familiar, and more helpful to the experimentalist capture is, the more routine it can become. Now, “easy” is the toughest design goal of all, and building general systems is hard and expensive. But what could we do in an afternoon or a few? I think the answer is quite a lot, provided the “we” is a joint effort of the experimentally and computationally inclined. Experimentalists must repeatedly show what they do, explain how they think, and critically test prototypes. Computationalists must repeatedly observe all the acts of experimentation, listen for unstated assumptions, and prototype the least intrusive, most experimentally efficient approaches. Together, both should maximize simplicity, flexibility, extensibility, and fun.

Capturing the experimental record at the source in real time in all types of laboratories will smooth the path to systems that automate capture and combine it with the extraction of provenance and annotatation of datasets. To reach the provenance infrastructure of the future that everyone needs, we need to understand the diversity of actual experimental practice and to start solving that most difficult problem of provenance capture. A brief dollop of altruism that focuses on very quick, lightweight, shareable improvements could immediately help experimentalists, provoke engagement across boundaries, and seed more sustainable collaborations. So, in the spirit of the rules for the provenance of computational experiments and instrument data [7,8], I offer ten simple rules for interdisciplinary collaborations on provenance capture. The order of the rules roughly parallels the workflow of discussions, from that first exploratory cup of coffee to the migration to grander schemes and bigger data. While each rule varies a bit in the distribution of tasks to experimentalists and computationalists, all require a joint effort. Our current practice is described in Box 1.

**Box 1. Our Practice So Far**

As a computational biologist who has also done maize genetics for the past nine years, I have the privilege of directly experiencing the realities of my experimentalist colleagues while watching how well—or not!—my computational ideas address the practical problems of experiments’ provenance. We study a set of ≈55 distinct maize mutants that produce necrotic or chlorotic lesions on leaf tissue [13,14]. We package selected seed for planting; plant in a field at research farms or in pots or trays in the greenhouse; repeatedly observe at least eight different phenotypes for each plant; pollinate with selected plants; photograph leaves in situ or ex situ; orally describe each mutant family; collect, lyophilize, and freeze samples of leaf tissue for DNA sequencing; harvest and shell pollinated ears; and file the corn for easy retrieval from the cold room. Field data are collected in the form of images (either standardized or free-form), spreadsheet tables, audio recordings, dumps of hourly weather data from a local recording station, and geographic coordinates of the first row of each field.

How do we apply the rules? Our simple provenance system for maize genetics has gradually evolved. The basic system was designed in the course of the first crop, with much discussion with maize colleagues about what and how they track their provenance. The result has proven very versatile and robust, needing only minimal changes despite the changing circumstances of each crop and the addition of other experiments and data and object types. Robustness is difficult to quantitate, but the system has so far managed approximately 5,900 families of maize (4,000 in active use), 18,000 images (including duplicates and test shots), 1,000 tissue samples, and 430,000 facts (including both primary data and reverse indices). We have added experiments and experimental protocols over the years, changed key equipment more than once, and worked with approximately 20 students so far in the project. These volumes are small compared to those of many experimental groups, especially in maize, but may suggest our experimental milieu.

The heart of our system is the unique identifiers. *Every* physical object that contributes directly to the production of our biological materials or for which data are collected has a unique identifier. There are many types of objects involved, and we often need to know what to do quickly, so we use mnemomic identifiers that distinguish each type of object and distinguish plants and their progency from equipment. Standard equipment for the field and seed room are not tracked—staplers, aprons, and shellers are all interchangeable. Cameras and scanners are not, so each has a distinctive name. So far, lenses are permanently assigned to cameras, and camera names are recorded as part of the photographic data. If we were to exchange lenses among cameras, it would be simple to name the lenses and their associations with cameras in the provenance system so that past and present data were correctly annotated.

Each plant is tagged with a sturdy barcoded paper strip that has multiple tear-off tags, each printed with the plant’s identifier, bar code, and an abbreviated symbolic genotype. The plant’s identifier becomes the primary key for all tabular data and seed from that plant, and it is the plant’s name in audio narratives, linking genotypes, phenotypes, samples, and data. Plant identifiers are 15 characters and state the year of planting, season, family number, inbred background if relevant, and the row and plant number for each plant. Redundancy is built into the identifier to help guard against information loss. Pollinations are labelled with tear-off tags from the plants serving as female and male for that particular cross. Stapled together and to the seed envelope, they identify the shelled seed for inventory, retrieval, and packing. The few person-days spent tagging the thousands of plants in each crop saves many person-months in data collection and verification, inventory management, and computation.

Pots, trays, seed packets, row stakes, tissue samples, boxes, sleeves, and seed bags all receive unique six-character identifying bar codes, with the identifiers for each type of object beginning with a single mnemomic letter. All letters, including those for inbred lines, are unique. Leaves are identified by either a relative or absolute coordinate on the plant, depending on the experiment. Apart from the leaf identifiers, all identifiers and their components are automatically generated, a lesson learned in the second year of field work when identifiers for a few families of siblings were inadvertantly duplicated.

Identifiers are printed in a large, bold font, along with their one-dimensional bar codes, on labels or tags. Labels and tags are generated with custom scripts and open-source code [9,10,15,16]. Our script collection includes code to generate individual tags to replace those with worn, illegible bar codes, hastily repurposed tags from sibling plants, and tags with retrospectively corrected data.

*Every* action or datum involving a barcoded object is recorded by scanning the bar code into a data table in a spreadsheet, either at the moment of the action or shortly thereafter. Contemporaneous data collection is one of our best safeguards against mangled data, permitting correction while the object or action is immediately present. It also helps us spot procedural bottlenecks and error-prone operations for process improvement. The only exception to the rule of contemporaneous data collection is for intermediate forms of the data, such as emerging lesion phenotypes or the pollination and photographic plan for a plant. These are stored on each plant as color-coded paper twist ties, with the date and initials of the human scorer stored on the first plant of the row. (We do record dates and scorer of each plant on it as needed, for example, when determining the onset of phenotypes.) These decisions can change as the phenotypes develop and pollinations proceed, so we usually record only the final evaluation or intention.

Most data are collected by scanning bar codes into a spreadsheet running on a tablet, using a matchbox-sized bluetooth scanner. Representative leaves from selected mutant plants are photographed to record phenotypes and to provide data for their quantitative characterization. Other photographs compare phenotypes among families and document surprises. Audio recordings of descriptions of the field, crop, families, and individual plants are collected throughout the field season, formerly with various dictaphone arrangements and now with the tablets. Their transcription lags, so we are now experimenting with speech-to-text programs.

Conversion of data from spreadsheet to database uses a family of Perl scripts and modules, including a library of regular expressions. As our data collection machinery has migrated from menu scanners to iPads and spreadsheets, and as students have come and gone, the characteristic errors that appear during data collection have changed. We dump the data as csv files and manually check those files before processing them and inserting their data into the database. Each student reviews the data he or she collected, and we also review each other’s data. We also perform different post hoc checks, depending on the operation—making sure each row and packet are accounted for at planting, that all recorded and unrecorded pollinations are harvested, that every ear used in pollinations is unique, etc.

Computationally, our provenance system uses a mix of tools: a declarative database for crop and data management, including pedigree computations; emacs org-mode, for the lab’s notebooks; git and tar for archiving ASCII data and code; and Perl scripts for generating tags and labels, generating org-mode tables with embedded, readily visible calculations, and for converting data from spreadsheet dumps and org files to the database. All types of files are backed up on two physically different RAID arrays. The experimental provenance system was developed before our system for computations and analyses, so the two interact at several levels without forming a monolith.

Images, files, and file and directory names all self-identify. Our leaf images include a barcoded tag from the plant, marked with the leaf number. (This practice has rescued data from scanning errors more than once.) All ASCII files begin with a string that includes the file’s full path. Files or data increments produced by code include comments specifying the name of the producing file or function, the source file for the data, and the timestamp of production. File names for audio recordings are descriptive now that we collect these with a tablet instead of a dictaphone. Names in directory trees are descriptive; camera and scanner names form part of the directory tree for primary data storage, helping us rapidly locate the data referenced in tables. Our lab notebook was formerly a set of physical notebooks and ASCII files. We recently switched to emacs org-mode, which facilitates project management and publication as well as written narratives. We photograph whiteboards, oddities, and paper, cross-referencing the images in our org-mode files and filing them in the same directories as the work they reference. We occasionally record conversations, and these are cross-referenced and filed in the same way.

Data and computation semantics reside in predicate and argument names and comments in files, but the more complex semantics still live in text files or my brain. Our next provenance frontier is to compute our metadata more easily, starting with experimental images intended for public deposition. Current ontologies denote only a tiny fraction of what we deem important, but this may change in the future.

## Rule 1. Go Sideways and Backward to Go Forward

What do the experimentalists track now, and what physical items and ideas interact with that? Similarly, many materials and methods have a history that is crucial to capture: genetic pedigrees and macromolecule preps are examples. So, discuss each major phase of the experimental lab’s life and how those phases relate to its work of today and tomorrow. Retrospectively entered data then join a consistent framework, rather than being kludged in.

## Rule 2. Improve the Acts of Experimentation

No one willingly adds encumberances, so changes have to produce a net gain over the entire experimental workflow. People are often willing to sacrifice a few person-days to save person-months, but to identify improvements is a joint labor of reengineering. Multiple interactions about and observations of the “same” experimental task show the ways in which the work varies, pinpointing process improvements and delimiting a design’s flexibility.

## Rule 3. It’s Gotta Beat a Spreadsheet

Spreadsheets are ubiquitous because they’re flexible, well understood by a large community (including students), great for prototyping an experimental workflow, and collect data simply. That combination is hard to beat! Yet, minor innovations in spreadsheets can yield big improvements in provenance. Examples include restructuring repeated free text descriptions as menu items and providing optional pop-up boxes with definitions of the lab’s terms and methods. More extensive systems with designer interfaces or back ends can be merited if the experimental workflows are very regular, but any proffered replacement should be as simple and as robust as a spreadsheet to use and maintain. Homebrew systems without a trivial maintenance path die once the graduate student who built them moves on.

## Rule 4. Barcode Everything Important and Keep the Labels Current

Each type of physical object or datum should have a distinctive, mnemomic identifier that tells you what it is without needing a reference guide or a gadget. What should be memorialized and how much information should be incorporated into the identifier for optimal tracking depends on the laboratory, and designing good identifier systems that are robust to change takes care. Mnemomic identifiers are easier to use in everyday experimental discourse than unadorned integers but may need more maintenance, especially in the face of the inevitable revisions. When the laboratory has many different types of physical objects and the context of their relationships is important in knowing what to do (usually quite urgently), then mnemomic identifiers can be a great help. They also have the advantage of letting one embed redundant information into the identifier. When the objects form relatively few types and the relationships among them are as yet unknown, then a centrally assigned integer can prove simpler in the long run. In either case, it is crucial to avoid embedding any (often subconscious) biological assumptions or inferences in the identifier. Similarly, storage systems and the organization of collections change over time. Rather than build fungible relationships and inferences into the identifier, identify the bone and the drawer it’s in today separately; store the encoded site of the bone’s collection in a database, revising the site’s coordinates when Global Positioning System (GPS) data are substituted for sunsights; and discover the relationships among bones collected at the same site by experiments.

Hastily improvised or newly inadequate identifiers are a fact of life and may not be transformed into the standard scheme for some time. If any part of the identifier for the object changes in the database (that site has more bones than we have characters in the identifier!), print the new identifier and its bar code on a label and attach it to the object so that both new and old labels can be read as needed. Keeping the labels current with the inevitable changes in the databases prevents the confusion that results from scanning old labels into new data schemes, minimizing repairs.

Once the identifier scheme is worked out, producing good labels is easy. Both open-source and commercial programs to generate bar codes are available, and it’s easy to write one’s own [9,10]. Print the identifier in large, bold font next to the bar code on sturdy label or tag stock so that a glance tells the story. There are a variety of materials, tags, and labels that are waterproof, take ink well without smearing, and tolerate temperature and humidity extremes so that plates and tubes can be barcoded.

## Rule 5. Make Everything Self-Identifying

Any bar code can be mangled on scanning or land in the wrong place in the spreadsheet. Looking at the physical artifact or data file can resolve these problems, but only if those things self-identify. Self-identification can be as simple as descriptive directory and file names, including file names as the first line in text files, or photographing a labelled container or rack of tubes. Though namespace collisions can occur with descriptive names, they are far more legible than random strings. A contemporaneous record guards against all errors except mislabelling the original.

## Rule 6. Use Version Control and Backups

Electronic laboratory notebooks are available in both open-source and commercial versions, and some may fit a group’s workflow well enough to justify the effort and expense of adoption. However, for many, a mnemomically named directory scheme, spreadsheets, text files, version control, and a RAID array may be enough—and a significant improvement. Version control of directories with ASCII data and notes is a cheap way to archive and time-stamp changes, emulating electronic notebooks while preserving workflow flexibility. It also provides the most insurance against fat fingers, fatigue, and forgetfulness. Calling export and backup scripts from a cron job or a big green “archive now!” button reduces human supervision.

## Rule 7. Automate Gluing over Cleaning

It’s usually not worth investing in sophisticated error correction much beyond essential reformatting. Many errors are idiosyncratic and disappear with practice in cleaning one’s data. Cleaning is painful, even with macros, but it teaches one to minimize collection errors, increases attention to experimental details, and creates another opportunity to check for lurking substantive errors. Rather, automate data transformations and archiving, and review characteristic errors from time to time. A similar principle applies to data generated in high-throughput facilities. If its managers are amenable, automating data transfers from a facility to the ultimate storage device saves time and error.

## Rule 8. Integrate the Paper

Many brains, even young ones, think more fluently in front of paper (or whiteboards) than screens. Photograph or scan these, cross-referencing the images in whatever the group uses for electronic notebooks and filing them in the appropriate directories. Groups that use just a few forms of stationary may benefit from switching to paper random-dot or grid notebooks and recording pens, but this may be too restrictive or expensive for others. Don’t forget the other form of “paper” that is produced by thinking out loud, and capture that with audio recordings. Common smartphone and tablet operating systems all run free voice recorder apps. Some groups may be able to use speech-to-text systems successfully to collect data.

## Rule 9. Prepare to Extract Metadata

Spreadsheet field headers, database attributes, and free text notes are crude metadata. Scooping them into a database that indexes their location will facilitate eventual extraction and lets one track other information more easily than recursive greps. The scoops need be no fancier than a text box on a form, a script that parses a csv or Excel file, or names of pdf files of paper notebook pages describing an experiment’s method [11]. More sophisticated tools that embed metadata collection in spreadsheet templates are also available [12]. Watch for repeated phrases, which are good metadata candidates.

## Rule 10. Only the Biologists Know for Sure

The harder part of metadata is ensuring the data’s actual and declared semantics match. The experimentalist is the only one who knows, and intermittent discussion will reveal crucial subconscious information. It follows that the definitions of metadata terms must be available at the moment of entry. A box for free text insertion lets one capture and analyze emerging needs, given a more committed collaboration.

Should you use existing standards, ontologies, and metadata (Box 2)? Of course, if these conventions are stable, capture what the experimentalist needs to say, and the experimentalist agrees with the convention's semantics. Ontologies in rapid-flux, fuzzily defined terms or odd lumpings of tradtional nomenclature are not good candidates for describing experiments. Then it is particularly important to record how the experimentalist describes the experiment and its data and to transmit that information to ontologists so they have more usages to study, while together you use them to define new metadata. As complex data accumulate in public resources, those resources will have to manage the migration of ontological terms. For now, free text seems the best guide to accurate migration, albeit the slowest.

**Box 2. A Glimpse of the Landscape**

An abyss separates the practices in many laboratories and research on the semantics and provenance of data and computations, workflow systems, and electronic notebooks and groupware. Beyond the simple rules, scalably bridging the divide will require connecting today’s achievements into flexible, transparent, and interoperable ecosystems of applications that meet *experimentalists’* felt needs. Of course, we have seen this landscape before: a visionary system that addressed many of these issues was the Worm Community System of the early 1990s [17,18]. Perhaps the following sketchy list can stimulate some collaborative spanning.

**Metadata and provenance**

In the rules, I emphasized provenance acquisition, skirted the formalization of metadata, and ignored provenance maintenance and reconciliation [19–26]. Archivists call information about the origin and semantics of experimental objects, computations, datasets, and analyses “descriptive metadata” (a book’s content), distinguishing them from the “administrative metadata” needed to use and manage resources (a book’s library record). However, many desirable applications would use both notions transparently. The early successes of the Dublin Core (administrative metadata), Gene Ontology, and macromolecular crystallographic information file (mmCIF) (the latter two, descriptive metadata) encouraged the idea that metadata would naturally arise from ontologies and be exchanged through the web [27–29]. The result was a flowering of standards, societies, ontologies, and working groups, each aimed at a particular slice of biology. Much of this work is now represented in database annotations, ontology and Semantic Web languages, and projects that foster and archive these materials [30–32]. Nonetheless, experimentalists treat annotations as read-only data and are largely unaware of the rest of the infrastructure. The *Nature* methods checklist is an interesting mix of metadata and location information [33,34]. It may further stimulate work on automated extraction of scientific, descriptive metadata—and validation of the output!—as is now done for bibliographic, administrative information [35–40]. We might even hope for a day when datasets and computations have unique identifiers, rather like DOIs, to facilitate building chains of provenance in both senses.

**Workflow systems**

Increasingly, wet-bench and computational work form an integrated whole, but current workflow systems address either a portion of the wet-bench work—such as LabView’s abilities to interconnect multiple instruments and their data—or computations [41]. At the moment, connections between the two sides mostly reside in the experimenter’s brain or in his or her notebooks. Research so far has been mainly on the essential technical details of organizing, tracking, and managing data, code, cycles, and storage [42–47]. Heroic efforts are made to stimulate adoption by each system’s notional user communities, but the need for heroism suggests that we should watch many experimentalists more carefully to uncover their desires and reservations.

**Electronic notebooks and groupware**

Cross literate programming, record keeping, project management, and text editors. Their offspring range from the free and open-source ipython notebook and emacs org-mode aimed at individual investigators, to commercial systems for groups, to cloud-based editors such as Evernote and SimpleNote, to wrangling group documents in wikis, GoogleDocs, or SharePoint [48–51]. They vary enormously in their support of all the desiderata mentioned, as do the needs of research groups for these good things. It may be time to consider how open architectures could form modular, niche environments that also ease the experimentalists’ journeys to thoughtful computation.
